# The relationship between mean arterial pressure and decreased glomerular filtration rate in rural areas of Northeast China

**DOI:** 10.1186/s12882-015-0115-4

**Published:** 2015-08-13

**Authors:** Hongmei Yang, Xiaofan Guo, Xingang Zhang, Zhao Li, Shasha Yu, Liqiang Zheng, Wenna Li, Ying Zhou, Yingxian Sun

**Affiliations:** Department of Cardiology, The First Hospital of China Medical University, 155 Nanjing North Street, Heping District, Shenyang, 110001 Liaoning China; Department of Clinical Epidemiology, Library, Shengjing Hospital of China Medical University, Shenyang, Liaoning China

**Keywords:** Mean arterial pressure, Decreased glomerular filtration rate, Relationship, Rural areas

## Abstract

**Background:**

Low mean arterial pressure (MAP) can cause low renal blood flow and damage the kidneys. However, in the general population, it remains unclear whether or not decline in renal function is related to MAP. The present study examined the relationship between MAP and decreased glomerular filtration rate(GFR) in participans aged ≥35 years from the Liaoning province of China.

**Methods:**

A total of 11345 representative individuals aged ≥ 35 years was selected and a cross-sectional survey was conducted from January 2012 to August 2013 to describe the gender-specific prevalence and factors associated with decreased GFR in rural areas of Liaoning Province.

**Results:**

Men with decreased eGFR (eGFR < 60ml/min per 1.73m^2^) were older, and had higher meanWC, systolic and diastolic BP, PP, MAP, total fasting glucose, LDL-C ,glyceride and uric acid levels and were current drinker/smoker at the baseline (all P < 0.05). Those with low education level, low income, low physical activity, low hemoglobin and HDL-C level had decreased eGFR (all P < 0.05). In women, the results were similar to those of men, but DBP and drinking status had no associations with the eGFR at the baseline (all P < 0.05). After adjustment for age, men with MAP of >112.2 mmHg versus ≤ 93.8 mmHg had ORs for decreased eGFR of 2.367 (95 % CI: 1.248 to 4.488) .After multivariable adjustment, an MAP of >112.2 mmHg versus ≤93.8 mmHg had an OR for decreased eGFR of 3.249 (95 % CI:1.394 to 7.575) in men, whereas in women, MAP was not associated with decreased eGFR.

**Conclusions:**

MAP was associated with decreased eGFR in men, while in women MAP was not associated with decreased eGFR. These findings provide some evidence that a different adaptive response to renal regulation may exist in males and females.

## Background

Chronic kidney disease (CKD) is recognized as a global public health problem ,and patients with CKD have a significantly increased risk of cardiovascular disease [1]. Mild renal insufficiency has been considered to be an independent risk factor for cardiovascular disease, and the correlation in people, in the absence of other cardiovascular risk factors has been confirmed[2]. Mean arterial pressure (MAP) is considered to be the perfusion pressure for the organs of the body. Low MAP can cause low renal blood flow (RBF) and damage the kidney [3]. However, this doesn’t mean that RBF increases as the MAP increases [4–7].

Recent interest has been aroused in the relationship of renal damage with arterial stiffness evaluated by the augmentation index (AIx) and blood pressure including central blood pressure (cBP) and peripheral blood pressure (pBP). Several studies have investigated the association of AIx, cBP and pBP with glomerular filtration rate (GFR), but they have obtained inconsistent results [8–11]. How MAP affects kidney function and gender differences in this mechanism remains unclear.

In the general population, is unknown how MAP influences the GFR? This study examined the relation between MAP and decreased GFR in participants aged ≥ 35 years from the Liaoning province of China. Further, we studied the relationship to see if there was a difference between the sexes.

## Methods

### Study population

The study was approved by the Ethics Committee of China Medical University (Shenyang, China). Liaoning Province is located in Northeast China. A representative sample of people aged ≥35 years was selected, and a cross-sectional survey was conducted from January 2012 to August 2013 to describe the sex-specific prevalence and factors associated with decreased GFR in rural areas of Liaoning Province. The study adopted a multi-stage, stratified random cluster-sampling scheme. In the first stage, three counties (Dawa, Zhangwu, and Liaoyang County) were selected from the eastern, southern, and northern regions of Liaoning province. In the second stage, one town was randomly selected from each county (a total of three towns). In the third stage, six to eight rural villages near each town were randomly selected (a total of 26 rural villages). Exclusion criteria for individuals in the present study included pregnancy, malignant tumor, or mental disorder. All the eligible permanent residents aged ≥35 years from each village were invited to join the study (a total of 14 016 participants). Of those, 11 956 participants responded and completed the study: the response rate was 85.3%. All procedures were performed in accordance with standard ethical principles. Written consent was obtained from all participants after they had been informed of the objectives, benefits, medical procedures and the confidentiality of personal information. For illiterate participants, we obtained written informed consent from their proxies. In this report, we used data obtained at baseline, and only participants with a complete set of data for the variables analyzed in the study were included, making a final sample size of 11 345 (5253 men and 6092 women).

### Data collection and measurements

Data were collected during a single clinic visit by trained doctors and nurses using a standard questionnaire by face-to-face interview. Before the survey was performed, all eligible investigators were invited to attend training. The training content included the purpose of this study, how to administer the questionnaire, the standard methods of measurement, the study procedures, and the importance of standardization. After the training a strict test was carried out, and only those who scored perfectly on the test could become investigators. During data collection, our inspectors received further instructions and support. There was a central steering committee with a subcommittee for quality control.

Data on demographic characteristics, lifestyle risk factors, dietary habits, family income, family history of cardiovascular disease and medical history of hypertension were obtained by interview with a standardized questionnaire. Educational level was divided into primary school or below, middle school, and high school or above. Family income was classified as ≤5000, 5000–20 000 and >20 000 CNY/year.

According to the American Heart Association protocol, the investigators performed blood pressure (BP) measurement three times for each participant at 2-min intervals after at least 5 min of rest, using a standardized automatic electronic sphygmomanometer (HEM-907; Omron), which had already been validated according to the British Hypertension Society protocol [12]. The participants were advised to avoid caffeinated beverages and exercise for at least 30 min before the measurement. During the measurement, the participants were seated with the arm supported at the level of the heart. The average of three BP measures was calculated and used in all analyses.

Weight and height were measured to the nearest 0.1 kg and 0.1 cm respectively, with the participants in lightweight clothing and without shoes. Waist circumference (WC) was measured (to the nearest 0.1 cm) at the umbilicus using a non-elastic tape, with the participants standing, at the end of normal expiration. BMI was calculated as weight in kilograms divided by the square of the height in meters.

Fasting blood samples were collected from all participants in the morning after at least 12 h of fasting. Blood samples were obtained from an antecubital vein into Vacutainer tubes containing EDTA. Fasting plasma glucose (FPG), total cholesterol (TC), low-density lipoprotein cholesterol (LDL-C), high-density lipoprotein cholesterol (HDL-C), triglyceride (TG), serum creatinine (Scr) and other routine blood biochemical indexes were analyzed enzymatically on an autoanalyzer. All laboratory equipment was calibrated, and blinded duplicate samples were used.

### Definitions

According to the JNC-8 report [13], hypertension is defined as SBP ≥140 mmHg and/or DBP ≥90 mmHg and/or the use of antihypertensive medications. According to the World Health Organization (WHO) criteria, BMI was categorized into three groups: normal (BMI <25 kg/m2), overweight (25 ≤ BMI <30 kg/m2) and obesity (BMI ≥30 kg/m2) [14]. Abdominal obesity was defined as WC ≥88 cm for females and WC ≥102 cm for males [15]. Dyslipidemia was defined according to the National Cholesterol Education Program-Third Adult Treatment Panel (ATP III) criteria [16]. High TC was defined as TC ≥6.21 mmol/L (240 mg/dL). Low HDL-C was defined as HDL-C < 1.03 mmol/L (40 mg/dL). High LDL-C was defined as LDL-C ≥4.16 mmol/L (160 mg/dL). High TG was defined as TG ≥2.26 mmol/L (200 mg/dL). Dyslipidemia was defined as having at least one of the disorders mentioned above. Diabetes mellitus (DM) and anemia were both diagnosed according to the WHO criteria: DM was defined as FPG ≥7 mmol/L (126 mg/dL) and/or being on treatment for diabetes; anemia was defined as a hemoglobin level <13.0 g/dL (<130 g/L) for men and <12.0 g/dL (<120 g/L) for women [17, 18]. Hyperuricaemia was determined as uric acid >422 μmol/L for men and >363μmol/L for women [19]. Drinking and smoking status were divided into current drinkers/smokers and nondrinkers/smokers. Physical activity included occupational and leisure-time physical activity. A detailed description of the methods has been presented before [20]. Occupational and leisure-time physical activity were merged and regrouped into three categories: (1) low—participants who reported light levels of both occupational and leisure-time physical activity; (2) moderate—participants who reported moderate or high levels of either occupational or leisure-time physical activity; and (3) high—participants who reported a moderate or high level of both occupational and leisure-time physical activity.

The dietary pattern was assessed using recall of foods eaten in the previous year. First, the participants were questioned on their average consumption of several food items per week. The reported consumption was quantified approximately in terms of grams per week (vegetable consumption: rarely = 3, <1000 g = 2, 1000–2000g = 1, ≥2000 g = 0; meat consumption, including red meat, fish, and poultry: rarely = 0, <250 g = 1, 250–500 g = 2, ≥500 g = 3). Subsequently, a special diet score (vegetable consumption score plus meat consumption score) was calculated for each participant (range 0–6). Higher values of the diet score indicate higher meat consumption and lower vegetable consumption and greater adherence to a Westernized diet, while lower values indicate adherence to the Chinese diet. Similar methods for calculating diet score could be found in the ATTICA study [21].

### Assessment of glomerular filtration rate

Fasting blood samples were collected as described under “Data collection and measurement”. Serum creatinine (Scr) was measured enzymatically on an autoanalyzer. The GFR was estimated using an equation originating from the CKD Epidemiology Collaboration (CKD-EPI) equation [22], which is more appropriate than the Modification of Diet in Renal Disease (MDRD) Study group equation [23]. Decreased GFR was defined as estimated GFR (eGFR) <60 ml/min/1.73 m2.

### Statistical analysis

Descriptive statistics were calculated for all the variables, including continuous variables (reported as mean values and standard deviations) and categorical variables (reported as numbers and percentages). Differences among them were evaluated using Student’s t-test, ANOVA, non-parametric tests or the χ2-test as appropriate. Multivariate logistic regression analyses were used to identify independent factors and associated comorbidities of decreased eGFR, with odds ratios (ORs) and corresponding 95 % confidence intervals (CIs) calculated. All the statistical analyses were performed using SPSS version 17.0 software, and P values <0.05 were considered to be statistically significant.

## Results

### Baseline characteristics of study population

Table 1 presents baseline characteristics of the participants according to the eGFR categories and sex. Men with decreased eGFR (eGFR <60ml/min per 1.73m2) had higher age, mean WC, systolic and diastolic BP, PP, MAP, total fasting glucose, LDL-C, glyceride and uric acid levels and were current drinker/smoker at the baseline (all P < 0.05). Those with low education level, low income, low physical activity, low hemoglobin and HDL-C level had decreased eGFR (all P < 0.05). In women, the results were similar to those of men, but DBP and drinking status had no association with the eGFR in women.

**Table 1:** Characteristics of subjects with eGFR ≥ 60 or eGFR < 60 by different genders

	Male			Female		
Characteristics	eGFR ≥ 60	eGFR < 60	*P* value	eGFR ≥ 60	eGFR < 60	*P* value
N	5165	88		5939	153	
Age (year)	54.13 ± 10.64	68.86 ± 10.46	<0.001	52.97 ± 10.05	68.73 ± 8.96	<0.001
MeanWC	83.71 ± 9.70	87.03 ± 10.34	0.004	81.17 ± 9.69	84.07 ± 10.30	<0.001
BMI (kg/m2)	24.72 ± 3.54	25.34 ± 3.77	0.168	24.85 ± 3.75	24.79 ± 3.91	1
Average SBP (mmHg)	143.30 ± 22.41	161.31 ± 26.31	<0.001	139.68 ± 23.80	154.23 ± 26.29	<0.001
Average DBP (mmHg)	83.66 ± 11.73	89.04 ± 16.33	0.002	80.49 ± 11.41	82.51 ± 14.78	0.11
PP (mmHg)	59.63 ± 16.34	72.27 ± 21.71	<0.001	59.19 ± 17.81	71.72 ± 20.84	<0.001
MAP (mmHg)	103.54 ± 14.14	113.13 ± 17.43	<0.001	100.22 ± 14.32	106.42 ± 16.71	<0.001
Race group (han, %)	4891,94.7	85,96.6	0.43	5633,94.8	150,98	0.076
Education			<0.001			<0.001
Primary school or below (n, %)	2139,41.4	55,62.5		3325,56	133,86.9	
Middle school (n, %)	2433, 47.1	26,29.5		2146,36.1	18,11.8	
High school or above (n, %)	593,11.5	7,8.0		468,7.9	2,1.3	
Income (CNY/year)			<0.001			<0.001
<=5000 (n, %)	669,13	28,31.8		668,11.2	39,25.5	
5000-20000 (n, %)	2780,53.8	43,48.9		3293,55.4	77,50.3	
>20000 (n, %)	1716,33.2	17,19.3		1978,33.3	37,24.2	
Current drinking status (n, %)	2376,46.0	13,14.8	<0.001	173,2.9	3,2.0	0.804
Current smoking status (n, %)	2976,57.6	26,29.5	<0.001	961,16.2	44,28.8	<0.001
History of stroke (n, %)	458,8.9	29,33	<0.001	480,8.1	29,19.0	<0.001
History of heart disease (n, %)	530,10.3	28,31.8	<0.001	914,18.4	63,41.2	<0.001
Physical activity			<0.001			<0.001
Mild (n, %)	1132,21.9	51,58.0		2092,35.2	99,64.7	
Moderate (n, %)	3746,72.5	35,39.8		154,59.2	41,26.8	
High (n, %)	287,5.6	2,2.3		332,5.6	13,8.5	
Laboratory parameters						
Fasting glucose (mmol/L)	5.94 ± 1.65	6.58 ± 2.58	0.013	5.85 ± 1.58	6.54 ± 2.26	<0.001
Total cholesterol (mmol/L)	5.16 ± 1.03	5.45 ± 1.47	0.109	5.28 ± 1.09	5.98 ± 1.70	<0.001
Triglyceride (mmol/L)	1.66 ± 1.67	1.86 ± 1.59	0.005	1.60 ± 1.32	2.24 ± 1.75	<0.001
LDL-C (mmol/L)	2.87 ± .079	3.12 ± 1.01	0.03	2.96 ± 0.83	3.33 ± 1.14	<0.001
HDL-C (mmol/L)	1.41 ± 0.42	1.21 ± 0.27	<0.001	1.41 ± 0.34	1.35 ± 0.41	0.002
Hemoglobin (g/L)	148.95 ± 17.96	138.89 ± 20.56	<0.001	130.22 ± 14.50	125.75 ± 16.14	<0.001
Uric acid	331.85 ± 81.25	447.51 ± 122.02	<0.001	252.94 ± 64.55	362.89 ± 94.43	<0.001

Data are expressed as the mean ± SD or as n (%)

*BMI* Body mass index, *WC* Waist circumference, *CNY* Chinese Yuan (1 CNY = 0.161 USD), *SBP* Systolic blood pressure, *DBP* Diastolic blood pressure, *LDL-C* Low-density lipoprotein cholesterol, *HDL-C* High-density lipoprotein cholesterol

### The prevalence of decreased eGFR by quartiles of MAP in men and women

**Fig. 1:**
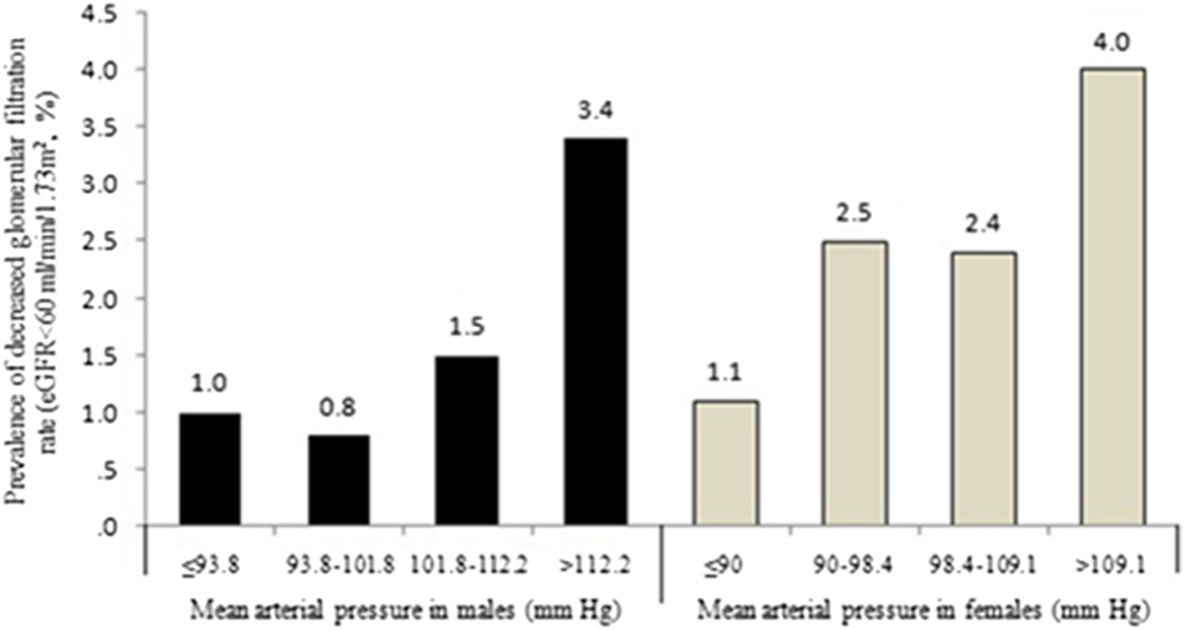
The prevalence of decreased eGFR by quartiles of MAP in men and women

Figure 1 shows that the prevalence of decreased eGFR by quartiles of MAP was different in men and women. For men, the prevalence of decreased eGFR increased as the MAP increased. The prevalence of decreased eGFR in men was lowest when 93.8 ≤ MAP < 101.8 mmHg, while for women, when 98.4 ≤ MAP < 109.1 mmHg, the prevalence of decreased eGFR was lower than in the group with lower MAP.

### The OR and 95%CI for the prevalence of decreased eGFR as a function of quartiles of MAP

**Fig. 2:**
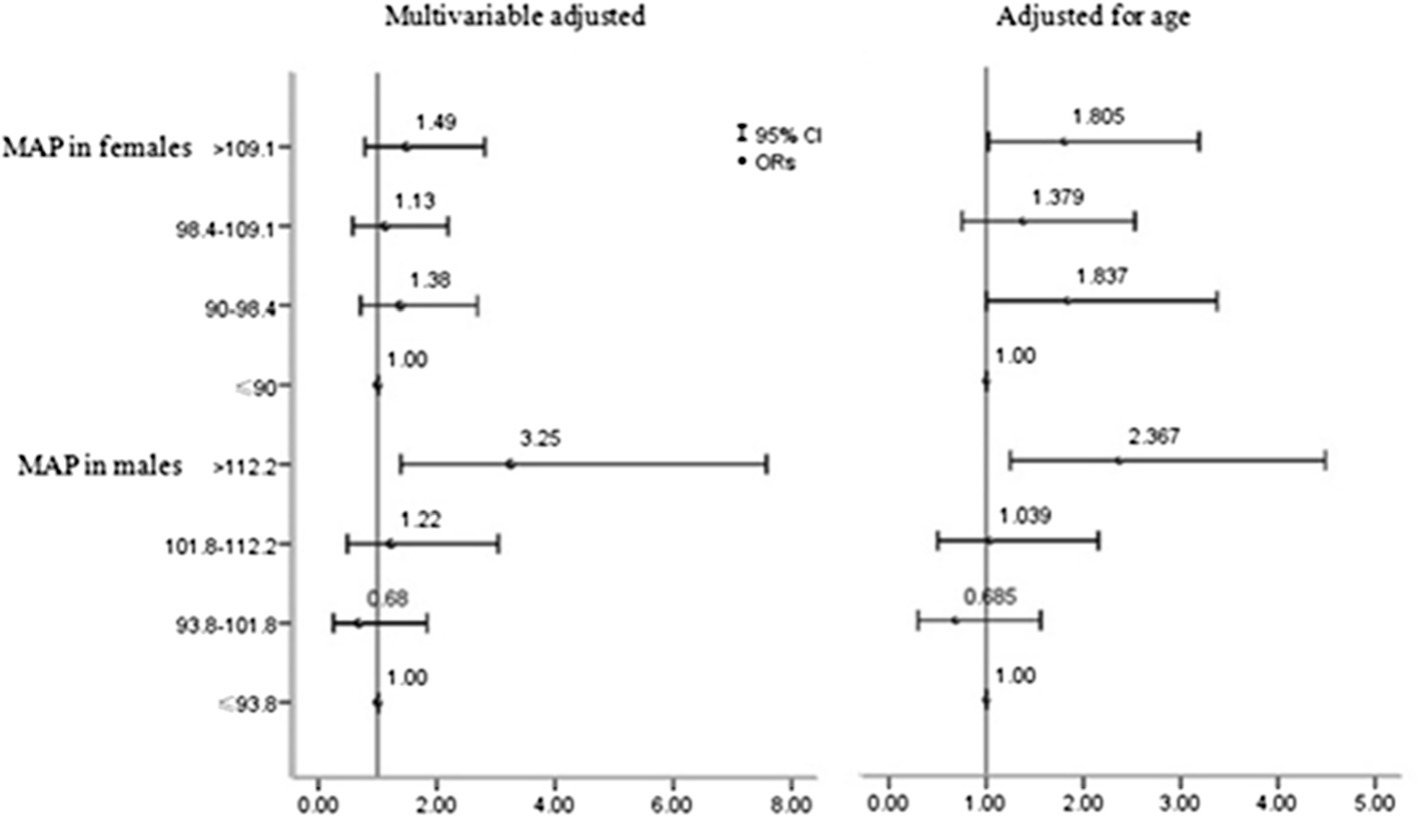
The OR and 95 % CI for the prevalence of decreased eGFR as a function of quartiles of MAP. Multivariable analysis adjusted for age, BMI, WC, mean SBP, mean DBP, mean PP, smoking status, drinking status, ethnic group, education, income, history of stroke, history of heart disease, total cholesterol, triglyceride, LDL-C, HDL-C, fasting glucose, hemoglobin, and uric acid

Figure 2 shows that after adjustment for age, that men with a MAP of >112.2 mmHg versus ≤93.8 mmHg had an OR for decreased eGFR of 2.367 (95 % CI: 1.248 to 4.488).After multivariable adjustment, men with a MAP of >112.2 mmHg versus ≤93.8 mmHg had an OR for decreased eGFR of 3.249 (95 % CI: 1.394 to 7.575). In women, MAP was not associated with decreased eGFR.

## Discussion

This is, to our knowledge, the first study to compare the association of MAP with decreased eGFR in a large group of participants. In this study, we demonstrated that MAP was associated with decreased eGFR in males. However, this association is not linear, from the lowest level of MAP to the highest level, but is significant only when MAP is >112.2 mmHg. It is well known that BP is characterized by its pulsatile and steady components. The pulsatile component, estimated by PP, represents BP variation and is affected by left ventricular ejection fraction, large-artery stiffness, early pulse wave reduction, and pulse rate [24, 25]. The steady component, estimated by MAP, is a function of left ventricular contractility, pulse rate, and vascular resistance and elasticity, averaged over time [26]. Adequate MAP is required to protect renal function, because below a certain MAP threshold, when autoregulation is exceeded, RBF decreases and this leads to acute renal injury [27]. When MAP does not exceed a certain level, there is no significant effect on renal function. With increasing MAP, the vascular resistance and elasticity increase, the arteries become progressively more stiff, and finally eGFR is decreased.

In our study, there were significant differences between men and women: in the latter, MAP was not associated with decreased eGFR. The trend of the prevalence of decreased eGFR as a function of quartiles of MAP appeared as a U-shaped curve. Recent studies have indicated that sex hormones play important roles in regulating the structure and/or function of the kidney, causing sex differences in a variety of characteristics [28–30]. Sex hormones affect the structure of the renal tubules and various aspects of renal functions in experimental animals and humans. Most of today’s knowledge is based on studies in rodents showing that sex-dependent phenomena in kidneys take place at the level of mRNA and/or protein expression of various transporters in the apical and basolateral membranes of the cells in specific parts of the nephron [31].

The limitation of this study should be considered in the light of the results. The study was cross-sectional, and therefore patients with decreased eGFR were identified retrospectively.

## Conclusions

In conclusion, MAP was associated with decreased eGFR in men, while in women MAP was not associated with decreased eGFR. These findings provide some evidence that a different adaptive response to renal regulation may exist in males and females, and paves the way for further studies aiming to identify the sex hormones involved in this mechanism.

## Abbreviations

eGFR: Glomerular filtration rate
CVD: Cardiovascular disease
MAP: Mean arterial pressure
PP: Pluse pressure
BP: Blood pressure
RBF: Low renal blood flow
BMI: Body mass index
WC: Waist circumference
FPG: Fasting plasma glucose
TC: Total cholesterol
LDL-C: Low-density lipoprotein cholesterol
HDL-C: High-density lipoprotein cholesterol
TG: Triglyceride
SBP: Systolic BP
DBP: Diastolic BP
WHO: World Health Organization
OR: Odds ratio
CI: Confidence interval
